# Supplementary material for: Total parasite biomass but not peripheral parasitaemia is associated with endothelial and haematological perturbations in Plasmodium vivax patients
Source: eLife. 2021 Sep 29;10:e71351. doi: 10.7554/eLife.71351 (PMC8536259; doi:10.7554/eLife.71351)
Supplement: Supplementary file 1. [file elife-71351-supp1.docx]

**Supplementary File 1: Topological analysis of the network graphs of healthy donors and *P. vivax* patients.**

| **Parameters** | **Healthy donors** | **Symptomatic Pv patients** | **P-value**  **(Pv vs HDs)** |
| --- | --- | --- | --- |
| Avg. number of neighbors | 12,296 | 7,103 | <0.0001 |
| Network diameter | 2 | 4 | <0.0001 |
| Network heterogeneity | 0.267 | 0.472 | <0.0001 |
| Network density | 0.473 | 0.267 | <0.0001 |
| Neighborhood Connectivity | 13.1 | 8.5 | <0.0001 |
| Avg. Shortest Path Length | 1.5 | 2 | <0.0001 |
| Betweenness Centrality | 0.02 | 0.04 | 0.04 |
| Closeness Centrality | 0.7 | 0.5 | <0.0001 |
| Number of Undirected Edges | 12.3 | 7.1 | <0.0001 |
| Topological Coefficient | 0.5 | 0.4 | 0.004 |
