# Supplementary material for: Total parasite biomass but not peripheral parasitaemia is associated with endothelial and haematological perturbations in Plasmodium vivax patients
Source: eLife. 2021 Sep 29;10:e71351. doi: 10.7554/eLife.71351 (PMC8536259; doi:10.7554/eLife.71351)
Supplement: Supplementary file 2. [file elife-71351-supp2.docx]

**Supplementary File 2: Oligonucleotides sequences used in the qRT-PCRs**

GAPDH -F: 5´- GACTGAGTGTGGCAGGGACT - 3´

GAPDH -R: 5´- GGCCTCCAAGGAGTAAGACC - 3´

IL-1A_F: 5′ - ATCAGTACCTCACGGCTGCT - 3′

IL-1A_R: 5′ - TGGGTATCTCAGGCATCTCC - 3′

IL-8_F: 5’ – TCTGCAGCTCTGTGTGAAGG - 3’

IL-8_R: 5’ - ACTTCTCCACAACCCTCTGC - 3’

ICAM-1_F: 5’- GGCCTCAGTCAGTGTGA - 3’

ICAM-1_R: 5’- AACCCCATTCAGCGTCA - 3’

ANG1_F: 5’- CAATGGGGGAGGTTGGACTGTA - 3’

ANG1_R: 5’- GAGGGATTTCCAAAACCCATTTTAT - 3’

ANG2_F: 5’- ACGTGAGGATGGCAGCGTT - 3’

ANG2_R: 5’- GAAGGGTTACCAAATCCCACTTTAT - 3’

ADAMTS13_F: 5’- CACAGGCCTCTCTTCACACA - 3’

ADAMTS13_R: 5’- GGTGTTAGGGGAGATGCTCA - 3’

SDC1_F: 5’- AGGACGAAGGCAGCTACTCCT - 3'

SDC1_R: 5'-TTTGGTGGGCTTCTGGTAGG - 3’

VEGF_F: 5’ - TGCAGATTATGCGGATCAAACC - 3’

VEGF_R: 5’ – TGCATTCACATTTGTTGTGCTGTAG - 3’

NOS3_R: 5’ - GTCGGAGCCATACAGGATT- 3’

NOS3_F: 5’ – CCAGCTAGCCAAAGTCACCAT - 3’
